# Lattice Thermal Conductivity of MgSiO_3_ Perovskite from First Principles

**DOI:** 10.1038/s41598-017-05523-6

**Published:** 2017-07-14

**Authors:** Nahid Ghaderi, Dong-Bo Zhang, Huai Zhang, Jiawei Xian, Renata M. Wentzcovitch, Tao Sun

**Affiliations:** 1 College of Earth Sciences, University of Chinese Academy of Sciences, Beijing, 100049 China; 2 Beijing Computational Science Research Center, Beijing, 100193 China; 3 Key Laboratory of Computational Geodynamics, Chinese Academy of Sciences, Beijing, 100049 China; 4 Materials Science and Engineering, Department of Applied Physics and Applied Mathematics, Columbia University, New York, NY 10027 USA; 5 Department of Earth and Environmental Sciences and Lamont-Doherty Earth Observatory, Columbia University, Palisades, NY 10964 USA

## Abstract

We investigate lattice thermal conductivity *κ* of MgSiO_3_ perovskite (pv) by *ab initio* lattice dynamics calculations combined with exact solution of linearized phonon Boltzmann equation. At room temperature, *κ* of pristine MgSiO_3_ pv is found to be 10.7 W/(m · K) at 0 GPa. It increases linearly with pressure and reaches 59.2 W/(m · K) at 100 GPa. These values are close to multi-anvil press measurements whereas about twice as large as those from diamond anvil cell experiments. The increase of *k* with pressure is attributed to the squeeze of weighted phase-spaces phonons get emitted or absorbed. Moreover, we find *κ* exhibits noticeable anisotropy, with *κ*_*zz*_ being the largest component and $({{\boldsymbol{\kappa }}}_{{\rm{\max }}}-{{\boldsymbol{\kappa }}}_{{\rm{\min }}})/\bar{{\boldsymbol{\kappa }}}$ being about 25%. Such extent of anisotropy is comparable to those of upper mantle minerals such as olivine and enstatite. By analyzing phonon mean free paths and lifetimes, we further show that the weak temperature dependence of *κ* observed in experiments should not be caused by phonons reaching ‘minimum’ mean free paths. These results clarify the microscopic mechanism of thermal transport in MgSiO_3_ pv, and provide reference data for understanding heat conduction in the Earth’s deep interior.

## Introduction

MgSiO_3_ perovskite (pv) is the most abundant mineral (80% in the pyrolite model) in the Earth’s lower mantle^1^. Its lattice thermal conductivity (*κ*) is under extensive investigation^2–11^ due to its critical role in understanding the dynamics and thermal evolution of the Earth. Experimental measurements of *κ* have been performed at room temperature from 0 to 144 GPa^4^ and from 473 to 1073 K at 26 GPa^3^, respectively. Theoretical simulations further extended the pressure (*P*) and temperature (*T*) range to that of the lower mantle (23 to 136 GPa, 2000 to 4000 K)^6–11^. These studies provide important information on the thermal transport in MgSiO_3_ pv, however, a consensus is yet to emerge on key issues such as the magnitude of *κ*, its *P* and *T* dependence, etc. Room temperature measurements using diamond anvil cell by Ohta *et al*.^4^ give relatively low *κ*: about 5.1 W/(m · K) at atmospheric pressure, 10.6 W/(m · K) at 31 GPa and 37.1 W/(m · K) at 144 GPa. Similar results were obtained by first-principles simulations by Dekura *et al*.^7^ and Stackhouse *et al*.^11^. In contrast, measurements with multi-anvil press by Manthilake *et al*.^3^ found much higher values: 15.6 W/(m · K) at 26 GPa and 473 K. If one extrapolates Manthilake *et al*.’s data to room temperature, the result will be nearly twice as large as that of Ohta *et al*. Such large *κ* are supported by classical molecular dynamics (MD) simulations by Haigis *et al*.^8^, whereas in conflict with other theoretical studies^7, 10, 11^. Besides the absolute magnitude, the *P* and *T* dependence of *κ* is also unresolved. Some studies indicate *κ* increases linearly with pressure^9^, while others found significant deviation from the linear dependence at high pressures^7^. Furthermore, standard phonon gas model (PGM) predicts *κ* at constant volume is inversely proportional to *T* in the classical high *T* limit^12^, but experimental measurements^3^ as well as MD simulations^8, 9, 11^ found much milder temperature dependence (*κ* ∝ *T* ^−0.43^). This anomaly in *κ*(*T*) has been attributed to phonons reaching ‘minimum’ mean free path (MFP) such that their contribution to *κ* saturates^13–15^. However a thorough analysis on the MFP and lifetimes of phonons in MgSiO_3_ pv has not been performed and the validity of this minimum MFP argument remains to be verified.

In general, theoretical investigations of lattice thermal conductivity of crystalline materials are conducted with two distinct approaches: MD and perturbative calculations based on PGM^16–19^. With the MD approach, one is able to treat the exact inter-atomic interactions, which can be important at high *T* where higher order (>3) anharmonic interactions become non-negligible^18^. The main drawback of MD is that only phonons whose wavelengths are commensurate to the simulation cell are present in the simulation. As a result, *κ* determined from MD depends on the size of the simulation cell (finite size effect)^20^. And due to constraints from computational resources, one may not be able to adopt sufficiently large simulation cells to minimize this effect. Also, in MD the atoms move as classical particles. This may introduce error when *T* is much lower than the Debye temperature and quantum effects are significant^21^. In contrast, PGM allows one to consider perturbatively how phonons get scattered by anharmonic interactions (usually truncated to the 3rd order) as well as impurities. The approximation (truncation) made on the inter-atomic interactions greatly simplifies the calculation and one is allowed to consider phonons of very long wave-lengths inaccessible to MD. Quantum effects can also be readily included in this formalism. PGM, combined with first-principles calculations of inter-atomic force constants^22^ and rigorous solution of the linearized Boltzmann transport equation (BTE) for phonons^23–25^, is now widely applied to determine the lattice thermal conductivities of crystalline materials. It works especially well in predicting *κ* near room temperature, where higher order (>3) anharmonic interactions are insignificant. Moreover, by examining the scattering rates (inverse of lifetimes) of individual phonons, one can gain useful insights into the microscopic mechanisms of thermal transport^26, 27^.

Here we take the perturbative approach based on PGM to determine *κ* of MgSiO_3_ pv from first principles. This is in line with previous studies by Dekura *et al*.^7^ and Tang *et al*.^10^. The important difference is that in the pioneering work by Dekura *et al*., only lifetimes of phonons at Γ point were evaluated explicitly, lifetimes of other phonons were obtained by extrapolation using an approximate relation between phonon frequencies and lifetimes. Also, third-order anharmonic force constants were computed via density functional perturbation theory for the primitive cell only. These approximations may introduce additional uncertainties in *κ* besides those inherent to PGM. Tang *et al*. followed a more rigorous procedure by computing the third-order force constants with a 2 × 2 × 2 supercell and evaluating lifetimes explicitly for all phonons. However, the *κ* they found is substantially lower than all other experimental and theoretical results, which is contrary to expectations. One of our aims is to resolve this discrepancy. Also, both Dekura *et al*. and Tang *et al*. employed the relaxation time approximation (RTA) to evaluate *κ* ^12^. RTA greatly simplifies the solution of BTE by omitting all the off-diagonal scattering terms. However the effectiveness of RTA is system-dependent^23, 28, 29^ and it is preferable to go beyond RTA and get the exact solution of BTE. We chose the ShengBTE code^24^, a well-developed package successfully applied to many materials, to perform the calculations. The dependences of *κ* on the range of anharmonic force constants and size of phonon **q**-point meshes were carefully examined to ensure good convergence. Intriguingly, the newly predicted *κ* at room temperature is significantly higher than previous first principles calculations^7, 10^ and in close agreement with multi-anvil press measurements^3^. Phonon lifetimes and MFP are then analyzed to understand the *P* and *T* dependence of *κ* microscopically. Finally we evaluate *κ* of MgSiO_3_ pv along typical geotherms of the lower mantle.

## Results and Discussion

**Figure 1:**
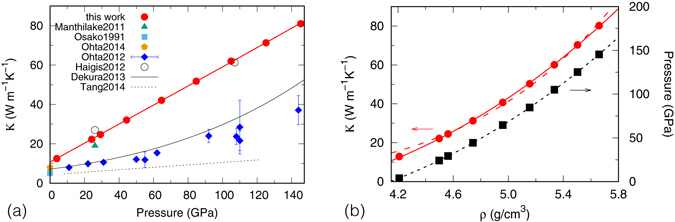
Lattice thermal conductivity of MgSiO_3_ pv at 300 K as a function of (**a**) pressure (**b**) density.

We first consider *κ* at 300 K, where higher order (>3) anharmonic interactions are insignificant and the perturbative approach based on PGM should work very well. We find *κ* of MgSiO_3_ pv is about 10.7 W/(m · K) at atmospheric pressure. It increases linearly with *P*, reaching 23.3 W/(m · K) at 26 GPa and 78.8 W/(m · K) at 140 GPa, as shown in Fig. 1(a). These values are about twice as large as the data of Ohta *et al*.^4^, whereas slightly higher than that of Manthilake *et al*.^3^. The linear *P* dependence of *κ* originates from the fact that *κ* exhibits very similar density (*ρ*) dependence as *P*. Indeed, we find *κ*(*ρ*) can be fitted nicely (solid line in Fig. 1(b)) by the Birch-Murnaghan equation as

1 $$\kappa (\rho )={\kappa }_{0}+\frac{3}{2}{\kappa }_{0}^{^{\prime} }[{(\frac{\rho }{{\rho }_{0}})}^{\mathrm{7/3}}-{(\frac{\rho }{{\rho }_{0}})}^{\mathrm{5/3}}],$$

where *ρ*_0_ is the equilibrium density at 0 GPa and 300 K, *κ*_0_ and ${\kappa }_{0}^{^{\prime} }\equiv {\rho \frac{d\kappa }{d\rho }|}_{{\rho }_{0}}$ are the thermal conductivity and density derivative of thermal conductivity at *ρ*_0_, respectively. To be specific, *ρ*_0_ = 4.15 g/cm^3^, *κ*_0_ = 10.7 W/(m · K), ${\kappa }_{0}^{^{\prime} }=121.6$ W/(m · K). An alternative form of *κ*(*ρ*) often seen in the geophysics literature is

2 $$\kappa (\rho )={\kappa }_{0}{(\frac{\rho }{{\rho }_{0}})}^{g}.$$

Fitting the calculated *κ* to this form yields the exponent *g* as 5.54 (dashed line in Fig. 1(b)), close to 5.6 found by Ohta *et al*.^4^. Interestingly, although our calculated *κ* are about twice as large as those of Ohta *et al*., their *ρ* dependence are quite similar.

**Figure 2:**
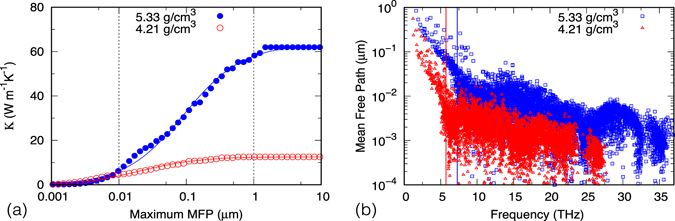
(**a**) Cumulative *κ* with respect to phonon mean free path at 300 K, (**b**) Mean free path versus phonon frequency at 300 K. Vertical lines in (**b**) correspond to frequencies of acoustic phonons at Brillouin zone boundary ${\bf{X}}\equiv (\frac{1}{2}00)$. Phonons reside on the left (right) side of the lines are predominately acoustic (optical). The corresponding pressures at *ρ* = 4.21 g/cm^3^ and 5.33 g/cm^3^ are 3.9 GPa and 104.9 GPa, respectively.

Figure 2(a) shows the cumulative *κ* with respect to phonon MFP. We see that major contributions to *κ* are from phonons with MFP between 0.01 to 1 *μ*m. At *ρ* = 5.33 g/cm ^3^ (*P* = 104.9 GPa), contributions from such phonons amount to ~84% of the total *κ*. For smaller *ρ* their contributions are less dominant, but still amount to ~63% of *κ* at *ρ* = 4.21 g/cm^3^ (*P* = 3.9 GPa). This is in contrast to good heat conductors like Si, where ~40% of *κ* at ambient condition comes from phonons with MFP greater than 1 *μ*m^30^. As shown in Fig. 2(b), phonons with MFP > 0.01 *μ*m are mostly acoustic modes plus a few low frequency optical modes. Overall they represent just a small fraction of all phonons. This indicates that *κ* of MgSiO_3_ pv is a sensitive quantity depending critically on the few phonons with long MFP: if the movement of such phonons somehow get hampered by extrinsic scattering such as impurities or grain boundaries, *κ* will drop considerably. (See Supplementary Information for a preliminary analysis on the effect of grain sizes). The relatively high *κ* from multi-anvil press measurement has long been a puzzle as most previous theoretical studies^7, 11^ found *κ* close to that of DAC measurement. Our results, which correspond to single crystals without defects, seem to indicate that the multi-anvil press results are closer to *κ* of pristine crystals. Besides sample conditions that may affect the measured *κ*, the specific experimental technique employed may also be relevant. In a more recent study^5^, Ohta *et al*. applied the microspot angstrom method to determine *κ* at ambient condition. The *κ* (~8 W/(m · K)) they found is 50% higher than that of Osako and Ito^2^ whereas close to their previous measurement at 11 GPa^4^ using the thermo-reflectance method. Further experiments are called for to fully resolve these discrepancies.

**Figure 3:**
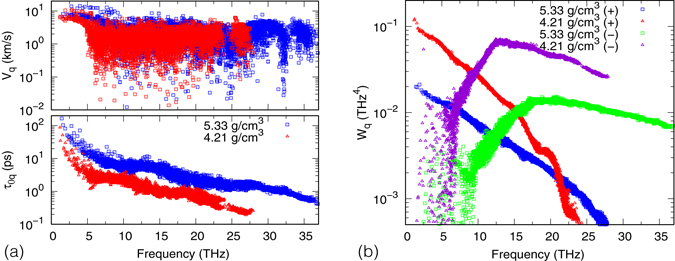
(**a**) Group velocity *v*_*q*_ and anharmonic lifetime *τ*_0*q*_ (**b**) Weighted phase space *W*_*q*_ versus phonon frequency at 300 K.

A characteristic feature of *κ* of MgSiO_3_ pv is that it increases more than 5 folds from 0 GPa to 100 GPa, as indicated by experiments as well as our calculations. Here we try to identify the microscopic origin of this increase. To simplify our analysis, we consider *κ* within the relaxation time approximation (RTA), which turns out to work well for MgSiO_3_ pv (within 1% of *κ*). Moreover, we ignore the relatively weak isotope scattering (1 to 3%). As such,

3 $${\kappa }_{0}=\frac{1}{3N{\rm{\Omega }}}\sum _{q}{C}_{q}{v}_{q}^{2}{\tau }_{0q},$$

where *C*_*q*_ and *v*_*q*_ are the heat capacity and group velocity of mode *q*, respectively; *τ*_0*q*_ is the anharmonic phonon lifetime, evaluated as

4 $$\mathrm{1/}{\tau }_{0q}=\frac{1}{N}\sum _{q^{\prime} q^{\prime\prime} }({{\rm{\Gamma }}}_{qq^{\prime} q^{\prime\prime} }^{+}+\frac{1}{2}{{\rm{\Gamma }}}_{qq^{\prime} q^{\prime\prime} }^{-}),$$

with ${{\rm{\Gamma }}}_{qq^{\prime} q^{\prime\prime} }^{\pm }$ being three-phonon scattering rates of absorption (+) and emission (−) processes. As *κ*_0_ depends on three quantities *C*_*q*_, *v*_*q*_ and *τ*_0*q*_, in the following we analyze how increasing *ρ* would affect each of them. A higher *ρ* gives rise to higher frequencies of phonons in MgSiO _3_ pv. Accordingly for a given *T* the phonon occupancies *n*_*q*_ become smaller, leading to a insignificant decrease in *C*_*q*_; On the other hand, the phonon group velocity *v*_*q*_ increases with *ρ* as phonons become more dispersive. However these increases are relatively mild (see the upper panel of Fig. 3(a)). For instance, in the long wavelength limit *v*_*q*_ become the velocities of elastic waves. From 0 GPa to 100 GPa, velocities of the longitudinal and transversal elastic waves increase ~30%^31^, much smaller than the 5-fold increase in *κ*. Therefore the increase in *κ* is mostly due to *τ*_0*q*_, as shown in the lower panel of Fig. 3(a). Recall *τ*_0*q*_ is determined by phonon adsorption (+) and emission (−) processes. For phonons of low frequencies, absorption (+) processes are the main scattering mechanism; For phonons of high frequencies, emission (−) processes dominate. A convenient way to see this is to consider the weighted phase space^26^

5 $${W}_{q}^{\pm }=\sum _{q^{\prime} q^{\prime\prime} }(\begin{array}{c}{n}_{q^{\prime} }-{n}_{q^{\prime\prime} }\\ {n}_{q^{\prime} }+{n}_{q^{\prime\prime} }+1\end{array})\frac{\delta ({\omega }_{q}\pm {\omega }_{q^{\prime} }-{\omega }_{q^{\prime\prime} })}{{\omega }_{q}{\omega }_{q^{\prime} }{\omega }_{q^{\prime\prime} }},$$

which corresponds to an approximation to 1/*τ*_0*q*_ with anharmonic interaction coefficients being identical for all scattering channels (see Eq. (7) in the Methods section). As shown in Fig. 3(b), ${W}_{q}^{\pm }$ decreases significantly at high *ρ* as phonons become more dispersive, leading to the near universal increases of *τ*_0*q*_ for all phonons. In principle, *τ*_0*q*_ are also affected by the anharmonic interaction coefficients, but their roles are likely to be secondary as changes in ${W}_{q}^{\pm }$ are already comparable in magnitude as those seen in *τ*_0*q*_. In summary, the increase of *κ* at high *ρ* is mostly due to the squeeze of the weighted phase space phonons may get emitted or absorbed.

**Figure 4:**
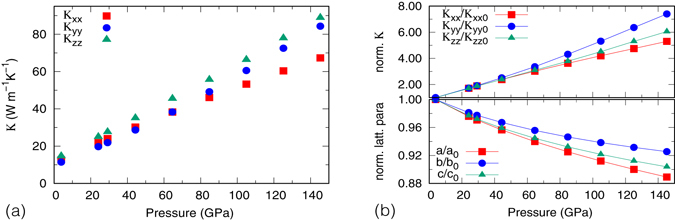
(**a**) Three diagonal components of *κ* as functions of pressure at 300 K; (**b**) Normalized conductivity *κ*_*αα*_/*κ*_*αα*0_ and lattice parameters as functions of pressure at 300 K.

For simplicity as well as easy comparison with experiments, so far we only considered the scalar averaged $\bar{\kappa }\equiv ({\kappa }_{xx}+{\kappa }_{yy}+{\kappa }_{zz})/3$. In general, for an orthorhombic crystal such as MgSiO_3_ pv the three diagonal components of *κ* are not identical. The extent of this anisotropy has not been resolved. Experimental measurements were limited to polycrystalline samples, therefore can not discern the anisotropy in *κ*. Theoretical calculations gave conflicting results. Ammann *et al*.^9^ found noticeable anisotropy in their non-equilibrium classical MD simulations whereas Stackhouse *et al*.^11^ reported that the anisotropy was within the error of their AIMD simulation. It is unclear whether such discrepancy is due to the different sizes of simulation cells or to the differences in the inter-atomic potentials. What we find in this study is that the absolute difference between the maximal (minimal) components of *κ* tensor, *κ*_m*ax*_(*κ*_m*in*_), increases with pressure, as shown in Fig. 4(a). But the relative magnitude of anisotropy is nearly pressure-independent: using $A\equiv ({\kappa }_{{\rm{\max }}}-{\kappa }_{{\rm{\min }}})/\bar{\kappa }$ as a measure for anisotropy, *A* = 24.1% near 0 GPa and 27.1% near 100 GPa. Such extent of anisotropy is comparable to those of upper mantle minerals such as olivine and enstatite^32^. Moreover, we find *κ*_*zz*_ is the largest among the three diagonal components in the pressure range we consider; *κ*_*yy*_ is the smallest at low *P*, but it increases the fastest with pressure and exceeds *κ*_*xx*_ near 60 GPa. The contrast in the *P* dependence of different *κ* components is most evident once they are normalized as *κ*_*αα*_/*κ*_*αα*0_, where the subscript “0”; represents the zero pressure, as shown in Fig. 4(b). Interestingly, *κ*_*αα*_/*κ*_*αα*0_ follow the same order as normalized lattice parameters: the larger normalized lattice parameter (smaller linear compressibility), the larger *κ*_*αα*_/*κ*_*αα*0_ along that axis. Note it is *κ*_*αα*_/*κ*_*αα*0_, not *κ*_*αα*_ itself, that exhibits a correspondence to linear compressibility. Whether such a correspondence is universal to all materials, or reserved to MgSiO_3_ pv, will be of interest for future studies.

**Figure 5:**
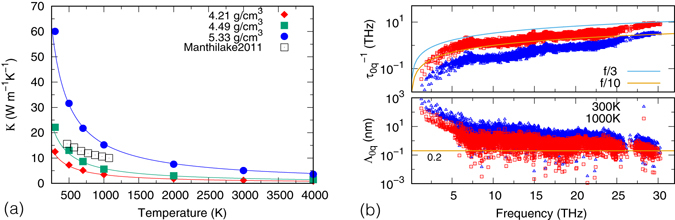
(**a**) Temperature dependence of *κ*. Lines are 1/*T* fits of *κ* in the range *T* ≥ 1000 K. Experiment by Manthilake *et al*.^3^ was conducted at 26 GPa (*ρ* ≈ 4.45 g/cm^3^). (**b**) Inverse of anharmonic lifetimes (${\tau }_{0q}^{-1}$) and mean free paths (Λ_0*q*_ ≡ *v*_*q*_ *τ*_0*q*_) versus phonon frequencies at *ρ* = 4.49 g/cm^3^. The calculated pressure for this density is 24.1 GPa at 300 K and 28.8 GPa at 1000 K. Lines with labels “f/3” and “f/10” in the upper panel denote 1/3 and 1/10 of the frequency, respectively. The horizontal line in the lower panel represents the inter-atomic distance 0.2 nm.

We now consider *κ* at elevated temperatures. Within standard PGM they are described by the same formula as *κ* at room temperature. In the high *T* classical limit, the number of phonons (*n*_*q*_ + 1/2) becomes *k*_*B*_ *T*/*ħω*_*q*_. Accordingly, *C*_*q*_ equals *k*_*B*_, *τ*_0*q*_ and *κ* at constant volume are inversely proportional to *T*; For lower *T* quantum effects are non-negligible and *κ* varies slightly faster than 1/*T* ^7^. Such *κ*(*T*) are in contrast to experimental measurements^3^ where much milder dependence were found. As shown in Fig. 5(a), the measured *κ* is close to our calculated value near room temperature, however the two deviate as *T* increases. At 1000 K they differ by about a factor of two. Note experiments were performed at constant pressure, thus the measured *κ*(*T*) also contains the effect of thermal expansion. But as the thermal expansivity of MgSiO_3_ pv is small (~2 × 10^−5^ K^−1^)^33^, the decrease in *κ* caused by thermal expansion is insignificant (~0.8 W/(m · K)). The large discrepancy between experiment and calculation is puzzling as the temperature we are considering is not particularly high: the maximal *T* reached in the experiment (1073 K) is close to the Debye temperature of MgSiO_3_ pv and about one third of the melting temperature (~3000 K at 26 GPa)^34^. The observed deviation from the 1/*T* law is commonly attributed^3, 8, 11^ to phonons reaching ‘minimum’ MFP^13–15, 35^. According to this theory, phonon MFP cannot be smaller than the inter-atomic distance (about 2.0 Å in MgSiO_3_). Once this limit is reached, MFP would not decrease further with *T* and *κ* saturates. To determine whether this is indeed the case, we analyze phonon MFP and lifetimes as shown in Fig. 5(b). We see even at *T* = 1000 K, the majority of phonons, especially the low frequency modes contributing most to *κ*, have MFP much longer than 2.0 Å. Therefore it is unlikely that the observed *κ*(*T*) is due to the minimum MFP. Moreover, recall MFP are products of phonon group velocities and lifetimes. In regions where phonon dispersions are flat, the group velocities are close to/equal zero. The corresponding MFP would be very short even at low *T* where anharmonic interactions are weak and phonons are well defined. Therefore to understand *κ* at high *T*, one should not focus on MFP alone, but to distinguish whether the short MFP are caused by intrinsically small group velocities, or short phonon lifetimes. If the lifetime is too short, say less than one vibrational period, then the phonon is not well defined and PGM may breakdown. For the present case, short MFP are mostly caused by small group velocities, as phonon lifetimes are at least 3 times longer than their periods at 1000 K. We therefore conclude phonons are well-defined and perturbation theory should work well under the experimental condition (from 473 to 1073 K at 26 GPa).

If the observed *κ*(*T*) is not due to minimum phonon MFP, then what is the culprit? In our view, radiative heat transport is a likely suspect. While thermal radiation is weak at room temperature, it increases rapidly with *T* (∝*T* ^3^) and its influence on *κ* may not be easily separated from those of lattice vibrations. Gibert *et al*. measured *κ* of olivine and found radiative heat transport accounts for 60% of total *κ* at 1120 K^36^. Similar effects may also be present in MgSiO_3_ pv. Besides thermal radiation, anharmonic heat flux may also play a role. As shown by Hardy^37^, heat flux in the standard PGM only corresponds to the diagonal part of harmonic heat flux. While this may well be sufficient at room temperature, contributions from anharmonic heat fluxes may become non-negligible at high *T* ^16, 18^. Indeed, for some model systems anharmonic fluxes were found^16, 18^ to contribute 40% of total lattice conductivity at half of the melting temperature. It will be interesting to explicitly calculate anharmonic heat fluxes in MgSiO_3_ pv and quantify their contribution to *κ*. However such endeavors are beyond the scope of the present study. For the moment, we simply assume standard PGM is valid for MgSiO_3_ pv at all temperatures. After all, precise predictions from PGM have their own merits and will serve as a benchmark for further investigations.

**Figure 6:**
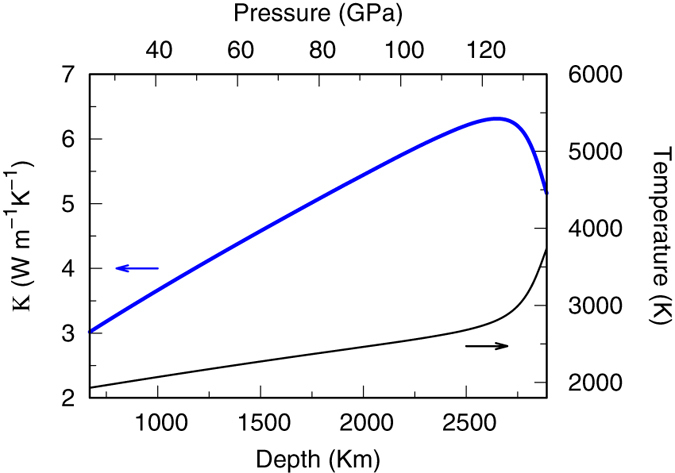
Lattice thermal conductivity of MgSiO_3_ pv in the Earth’s lower mantle predicted from standard PGM.

With *κ*(*P*, *T*) at hand, we now determine *κ* of MgSiO_3_ pv in Earth’s lower mantle. Figure 6 shows *κ* as a function of depth along typical geotherms of the lower mantle^1^. Increasing depth is accompanied by rising both temperature and pressure. The former will decrease *κ* whereas the latter increases it, and eventually *κ* is determined by these two competing factors. From 660 km to 2500 km, *κ* increases with depth, indicating the effect of pressure dominates. Near the core-mantle boundary temperature increases rapidly, and we see a decrease in *κ*. At the core-mantle boundary, *κ* is about 5.2 W/(m · K). Heat transfer in the mantle is dominated by convection. But in regions where mass transport is impeded (e.g. core-mantle boundary), conduction is the main mechanism. The knowledge we have on *κ* of MgSiO_3_ pv will help constrain the total heat flux between the core and the mantle, as well as to understand thermal evolution of the Earth.

## Methods

A central quantity for studying heat transport in PGM is the phonon distribution function *N*_*q*_^12^. Here the subscript *q* is shorthand for phonon wave vector **q** and branch index *s*. Accordingly, phonon frequency and group velocity are denoted as *ω*_*q*_ and **v**_*q*_. For systems with homogenous temperature *T*, *N*_*q*_ equals the equilibrium phonon occupancy ${n}_{q}\equiv 1/({e}^{\hslash {\omega }_{q}/{k}_{B}T}-1)$. For systems under a small temperature gradient ∇*T*, *N*_*q*_ deviates from *n*_*q*_, resulting in a net heat flux ${\bf{J}}=\frac{1}{N{\rm{\Omega }}}{\sum }_{q}\hslash {\omega }_{q}({N}_{q}-{n}_{q}){{\bf{v}}}_{q}$. The prefactor *N* is the number of unit cells in the system, Ω is the unit cell volume. The leading term in *N*_*q*_ − *n*_*q*_ is proportional to ∇*T* and, as **J** = −*κ* · ∇*T* (Fourier’s law), one can readily compute the thermal conductivity tensor *κ* once *N*_*q*_ − *n*_*q*_ is known.

Assume ${N}_{q}-{n}_{q}=-\frac{{C}_{q}}{\hslash {\omega }_{q}^{2}}{{\bf{E}}}_{q}\cdot \nabla T$, where ${C}_{q}\equiv {n}_{q}({n}_{q}+\mathrm{1)}{\hslash }^{2}{\omega }_{q}^{2}/{k}_{B}{T}^{2}$ is the mode heat capacity, **E**_*q*_ is an auxiliary function to be evaluated^24^, the BTE for *N*_*q*_ − *n*_*q*_ can be reformulated into linear equations for **E**_*q*_ as

6 $$\begin{array}{rcl}{\omega }_{q}{{\bf{v}}}_{q} & = & \frac{1}{N}[\sum _{q^{\prime} q^{\prime\prime} }{{\rm{\Gamma }}}_{qq^{\prime} q^{\prime\prime} }^{+}({{\bf{E}}}_{q}+{{\bf{E}}}_{q^{\prime} }-{{\bf{E}}}_{q^{\prime\prime} })\\  &  & +\frac{1}{2}\sum _{q^{\prime} q^{\prime\prime} }{{\rm{\Gamma }}}_{qq^{\prime} q^{\prime\prime} }^{-}({{\bf{E}}}_{q}-{{\bf{E}}}_{q^{\prime} }-{{\bf{E}}}_{q^{\prime\prime} })\\  &  & +\sum _{q^{\prime} }{{\rm{\Gamma }}}_{qq^{\prime} }({{\bf{E}}}_{q}-{{\bf{E}}}_{q^{\prime} })],\end{array}$$

where ${{\rm{\Gamma }}}_{qq^{\prime} q^{\prime\prime} }^{\pm }$ are three-phonon scattering rates of absorption (+) and emission (−) processes, expressed as

7 $${{\rm{\Gamma }}}_{qq^{\prime} q^{\prime\prime} }^{\pm }=\frac{\hslash \pi }{4}(\begin{array}{c}{n}_{q^{\prime} }-{n}_{q^{\prime\prime} }\\ {n}_{q^{\prime} }+{n}_{q^{\prime\prime} }+1\end{array})\frac{\delta ({\omega }_{q}\pm {\omega }_{q^{\prime} }-{\omega }_{q^{\prime\prime} })}{{\omega }_{q}{\omega }_{q^{\prime} }{\omega }_{q^{\prime\prime} }}{|{V}_{qq^{\prime} q^{\prime\prime} }^{\pm }|}^{2},$$

with ${V}_{qq^{\prime} q^{\prime\prime} }^{\pm }$ representing anharmonic interaction coefficients, ${{\rm{\Gamma }}}_{qq^{\prime} }$ is the scattering rate of isotopic disorder. This set of linear equations for **E**_*q*_ is solved iteratively^38^ in $\mathrm{ShengBTE}$. Thermal conductivity tensor *κ*_*αβ*_ is then expressed as

8 $${\kappa }_{\alpha \beta }=\frac{1}{N{\rm{\Omega }}}\sum _{q}\frac{1}{{\omega }_{q}}{C}_{q}{v}_{q\alpha }{E}_{q\beta }\mathrm{.}$$

The scalar average $\bar{\kappa }=\frac{1}{3}{\sum }_{\alpha }{\kappa }_{\alpha \alpha }=\frac{1}{3N{\rm{\Omega }}}{\sum }_{q}{C}_{q}{v}_{q}{{\rm{\Lambda }}}_{q}$, where ${{\rm{\Lambda }}}_{q}\equiv \frac{{{\bf{E}}}_{q}\cdot {{\bf{v}}}_{q}}{{v}_{q}{\omega }_{q}}$ is the phonon mean free path.

The right hand side of Eq. (6) contains both diagonal and off-diagonal scattering terms. The latter couple **E**_*q*_ with ${{\bf{E}}}_{q^{\prime} }$ and ${{\bf{E}}}_{q^{\prime\prime} }$, making the solution of Eq. (6) cumbersome. If one ignores all the off-diagonal terms, Eq. (6) becomes

9 $$\begin{array}{rcl}{\omega }_{q}{{\bf{v}}}_{q} & = & \frac{1}{N}(\sum _{q^{\prime} q^{\prime\prime} }{{\rm{\Gamma }}}_{qq^{\prime} q^{\prime\prime} }^{+}+\frac{1}{2}\sum _{q^{\prime} q^{\prime\prime} }{{\rm{\Gamma }}}_{qq^{\prime} q^{\prime\prime} }^{-}+\sum _{q^{\prime} }{{\rm{\Gamma }}}_{qq^{\prime} }){{\bf{E}}}_{q}\\  & = & \frac{{{\bf{E}}}_{q}}{{\tau }_{q}}\mathrm{.}\end{array}$$

This so-called relaxation time approximation (RTA) makes **E**_*q*_ independent from each other and easy to solve. With phonon relaxation time *τ*_*q*_ defined as ${\tau }_{q}^{-1}\equiv \mathrm{1/}N({\sum }_{q^{\prime} q^{\prime\prime} }{{\rm{\Gamma }}}_{qq^{\prime} q^{\prime\prime} }^{+}+\frac{1}{2}{\sum }_{q^{\prime} q^{\prime\prime} }{{\rm{\Gamma }}}_{qq^{\prime} q^{\prime\prime} }^{-}+{\sum }_{q^{\prime} }{{\rm{\Gamma }}}_{qq^{\prime} })$, **E**_*q*_ = *ω*_*q*_ *τ*_*q*_ **v**_*q*_ and ${\kappa }_{\alpha \beta }=\frac{1}{N{\rm{\Omega }}}{\sum }_{q}{C}_{q}{v}_{q\alpha }{v}_{q\beta }{\tau }_{q}$.

To get *κ* from Eqs (6–8), one needs to first know the harmonic and third-order force constants, from which *ω*_*q*_, **v**_*q*_, ${{\rm{\Gamma }}}_{qq^{\prime} q^{\prime\prime} }^{\pm }$ and ${{\rm{\Gamma }}}_{qq^{\prime} }$ are determined. For polar crystals like MgSiO_3_ pv, one also needs to know the Born effective charges and dielectric constants to deal with long-range dipole-dipole interactions^39^. Calculations of these quantities were performed with the projector-augmented wave (PAW) method^40^ as implemented in the Vienna ab initio simulation package (VASP)^41^. The electron-electron exchange-correlation interaction was described with the local density approximation (LDA)^42^. A 2 × 2 × 2 supercell containing 160 atoms was employed and a 2 × 2 × 2 Monkhorst-Pack mesh^43^ was used for Brillouin zone sampling. The plane-wave cutoff for electron wave-functions was set to 550 eV. We considered a series of densities, with static pressure *P*_0_ ranging from 0 to 140 GPa. At each density, harmonic force constants, Born effective charges and dielectric constants were determined with density functional perturbation theory^44, 45^. Third order force constants were obtained by first displacing atoms along symmetrically inequivalent directions, then analyzing the changes in atomic forces with respect to displacements. This finite-difference approach requires highly accurate atomic forces, hence a tight threshold (10^−7^ eV) was adopted for computing the electronic eigenfunctions. Once all the needed information were in place, they were fed into the ShengBTE code to compute *κ*.

The procedures as described above are all standard. Still, to get well-converged *κ* two things demand special care: (i) the range of anharmonic interactions, (ii) the size of **q**-point mesh for evaluating Eqs (6–8). We first consider (i). Anharmonic interactions are usually short-ranged. Thus to save computational efforts it is common to set a cutoff when computing third order force constants. This cutoff should not be too small, otherwise the effects of anharmonic interactions may not be fully accounted for. Yet an unnecessarily large cutoff may only increase the computational costs without bringing much improvement in accuracy. After intensive tests, a cutoff of 4.0 Å was chosen for all densities. This is a rather conservative choice, as we found results from a smaller cutoff (3.5 Å) are already quite reasonable (the changes in *κ*_*αα*_ are within 4%). Mode Grüneisen parameters derived from these third order force constants^24^ agree well with those from direct finite differences^46^, demonstrating the accuracy of these force constants. The averaged Grüneisen parameter is 1.48 at *ρ* = 4.21 g/cm^3^(*P*_0_ = 0 GPa), 1.08 at *ρ* = 5.33 g/cm^3^ (*P*_0_ = 100 GPa), in good agreement with previous studies (1.44 and 1.12)^3^. Moreover, we repeated the calculation at *P*_0_ = 100 GPa with a 3 × 3 × 3 supercell and a cutoff of 5.0 Å. The change in *κ*_*αα*_ is less than 2.5%. This shows that the 2 × 2 × 2 supercell and 4.0 Å cutoff are indeed sufficient for describing anharmonic interactions in MgSiO_3_ pv. We now consider (ii). The size of **q**-point mesh affects *κ* in two respects. It determines the types of phonons in the system, and the accessible scattering channels through which these phonons get emitted or absorbed. Therefore it is crucial to investigate the dependence of *κ* on the size of **q**-point mesh. We found *κ* evaluated on coarse meshes (e.g. 3 × 3 × 3) differ significantly (more than 50% in *κ*_*αα*_ components) from those on denser meshes (e.g. 8 × 8 × 8). But once the mesh is sufficiently dense, *κ* becomes insensitive to further increases in mesh sizes. To ensure good convergence in *κ*, we chose a 8 × 8 × 8 **q**-point mesh which is equivalent to a supercell containing 10240 atoms. Coarse meshes correspond to small supercells with hundreds of atoms, as typically employed in MD simulations. Since the difference between *κ* from coarse and dense **q**-meshes is very large, MD simulations with small supercells would suffer significant finite size effects and cannot give well-converged *κ*. For evaluating *κ* at relatively low *T* where higher order (>3) anharmonic interactions are insignificant, the perturbative approach based on PGM is more appropriate.

In theoretical calculations, it is handy to compute *κ* at different temperatures while keeping the density fixed. But in many applications knowing *κ*(*P*, *T*) is more convenient. To get *κ*(*P*, *T*), we first determined the thermal equation of state *ρ*(*P*, *T*) using the standard quasi-harmonic approximation (QHA)^46, 47^, then substituted it into *κ*(*ρ*, *T*) to get *κ*(*ρ*(*P*, *T*), *T*). QHA has long been applied to predict the structural parameters^48^, thermal equation of state^33^, as well as thermal elasticity^31, 49^ of MgSiO_3_ pv. Its effectiveness for this system is now firmly established^47, 50^. In particular, the deviatoric thermal stresses were found to be less than 0.2 GPa at 300 K and about 2 GPa at conditions of the Earth’s core-mantle boundary (*P* = 135 GPa, *T* = 4000 K)^49^, therefore the pressure predicted by QHA can be regarded as hydrostatic, just like the static pressure *P*_0_. For a given *ρ*, *P* and *P*_0_ are close (within 3 to 5 GPa) at room temperature. Their difference grows with *T* and reaches about 34 GPa at core-mantle boundary.

### Data Availability

The data for this paper are available from T.S. (tsun@ucas.ac.cn).

## Electronic supplementary material

Supplementary Information
