# Supplementary material for: Lattice Thermal Conductivity of MgSiO3 Perovskite from First Principles
Source: Sci Rep. 2017 Jul 14;7:5417. doi: 10.1038/s41598-017-05523-6 (PMC5511206; doi:10.1038/s41598-017-05523-6)
Supplement: Supplementary file 1 — Supplementary Information [file 41598_2017_5523_MOESM1_ESM.pdf]

Lattice Thermal Conductivity of $MgSiO_3$ Perovskite from First Principles (Supplementary Information)

Nahid Ghaderi, Dong-Bo Zhang, Huai Zhang, Jiawei Xian,
Renata M. Wentzcovitch, Tao Sun

1. Crystal structures and phonon dispersions

Table S1: Static structural parameters of $MgSiO_3$ perovskite (pv) at 0 GPa. $MgSiO_3$ pv has space group of $Pbnm$, and fractional coordinates that are not determined by symmetry are shown below.

	Expt.[a]	Static(this study)	Static[b]
a (Å)	4.7787(4)	4.732	4.711
b(Å)	4.9313(4)	4.895	4.880
c(Å)	6.9083(8)	6.841	6.851
V(Å^3)	162.80	158.46	157.50
x(Mg)	0.5141(1)	0.5159	0.5174
y(Mg)	0.5560(1)	0.5591	0.5614
x(O1)	0.1028(2)	0.1055	0.1128
y(O1)	0.4660(2)	0.4647	0.4608
x(O2)	0.1961(1)	0.1952	0.1928
y(O2)	0.2014(2)	0.2005	0.1995
z(O2)	0.5531(1)	0.5544	0.5582

a. Measurements from Horiuchi et al.[1]
b. Calculations by Wentzcovitch et al. [2]

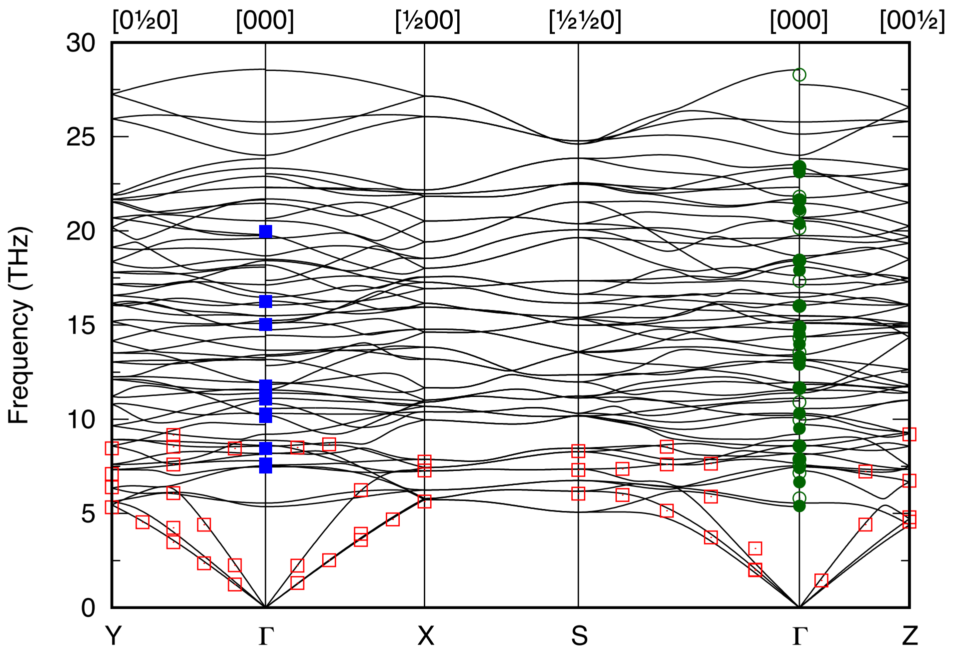

(a)

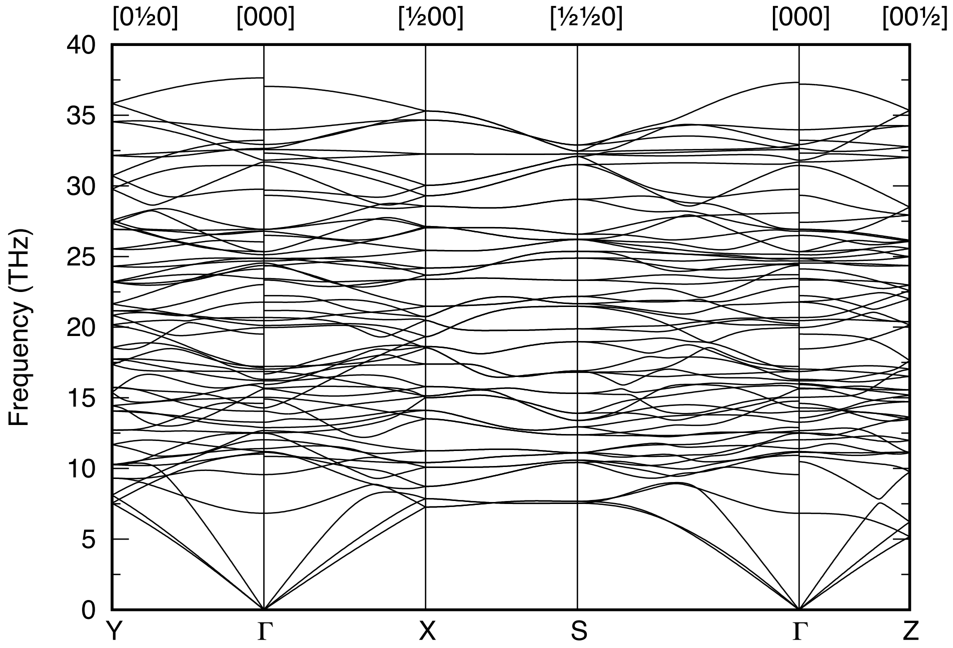

(b)

Figure S1. Phonon dispersions of $MgSiO_3$ perovskite: (a) $\rho = 4.21$ g/cm^3(P_0=0 GPa) (b) $\rho = 5.33$ g/cm^3 (P_0=100 GPa). Experimental data are shown as points: red open square: inelastic X-ray scattering measurements[3]; blue filled square: Raman spectroscopy[4]; green filled(open) circles: Infrared measurements on TO(LO) phonons[5] .

2. Effect of grain sizes on thermal conductivity of $MgSiO_3$ pv

A noticeable difference between experiments by Ohta et al. and Manthilake et al. are the samples' grain size. In the diamond anvil cell experiment by Ohta et al., the average grain size of the sample is relatively small($\sim 1\ \mu$m), whereas the multi-anvil press experiment by Manthilake et al. employed larger samples with grain sizes 10 to 15 μm. In the following we present a preliminary analysis on the effect of grain sizes using the grey model(or Casimir limit).

In this model, κ is evaluated with RTA as $\kappa = \frac{1}{3N\Omega}\sum_q C_q v_q^2 \tau_q$, where phonon lifetime τ is determined according to Matthiessen's rule as $\tau^{-1} = \tau_{ph}^{-1} + \tau_B^{-1}$, with τ_{ph}^{-1} being scattering rate from anharmonic phonon-phonon interaction and τ_B^{-1} from grain boundaries. For each mode, τ_{ph}^{-1} is determined from ShengBTE calculations, $\tau_B^{-1} = v_g/Fd$, where v_g is the phonon group velocity, d is the grain size, F is the form factor characterizing the width to length ratio of the grain. Following Fugallo et al.,[6] we set $F = 0.5$.

From Fig. S2 we see the effect of grain size is most prominent at high pressures. Near 100 GPa, κ of samples with grain size 1 μm is smaller than that of 10 μm by ~25%. Near 26GPa, the difference is about 13%. Similar variations of κ with grain sizes has been observed experimentally in other systems such as SnO_2, where κ drops from ~40 W(m·K) to ~25 W(m·K) when grain size decreases from 8 μm to 2 μm.[7]

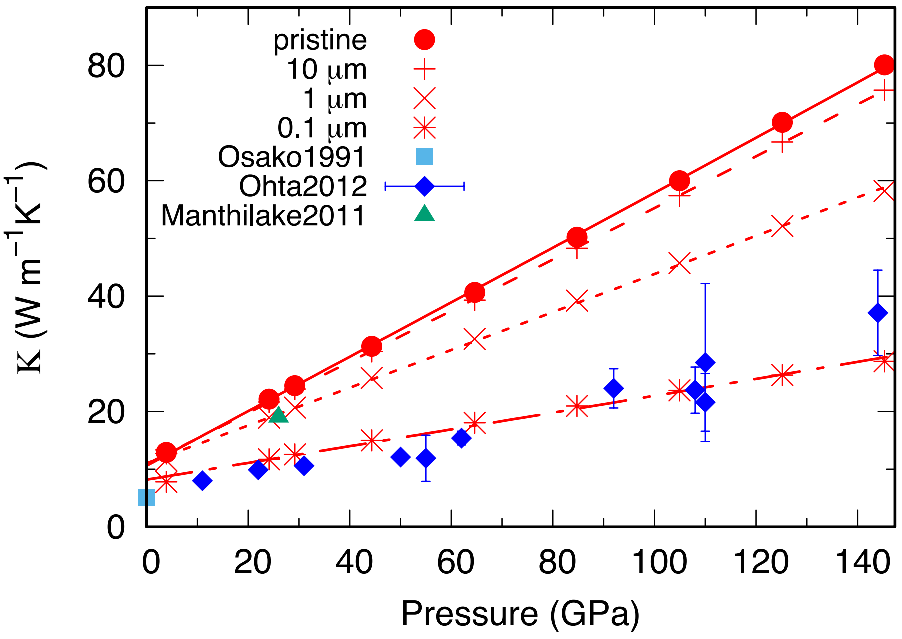

Figure S2. Lattice thermal conductivity of $MgSiO_3$ pv with different grain sizes.

References:

[1] Horiuchi, H., Ito, E. & Weidner, D., Perovskite type $MgSiO_3$: single crystal X-ray diffraction study. *Am. Mineral.*, **72**, 357-360 (1987).

[2] Wentzcovitch R. M., Ross, N. L. & Price G. D. Ab initio study of $MgSiO_3$ and $CaSiO_3$ perovskites at lower-mantle pressures. *Phys. Earth Planet. Int.*, **90**, 101-112 (1995).

[3] Wehinger, B. et al, Dynamical and elastic properties of $MgSiO_3$ perovskite. *Geophys. Res. Lett.*, **43**, 2568-2575 (2016)

[4] Durben, D. J. & Wolf, G. H. High-temperature behavior of metastable $MgSiO_3$ perovskite: A Raman spectroscopic study. Am. Mineral. , **77**, 890-893 (1992)

[5] Lu, R., Hofmeister, A. M. & Wang Y., Thermodynamic properties of ferromagnesium silicate perovskites from vibrational spectroscopy. *J. Geophy. Res.,* **99**, 11795-11804 (1994)

[6] Fugallo, G., Lazzeri, M., Paulatto, L. & Mauri, F. Ab initio variational approach for evaluating lattice thermal conductivity. *Phys. Rev. B* **88**, 045430 (2013)

[7] Fayette, S., Smith, D. S., Smith, A. & Martin, C. Influence of grain size on the thermal conductivity of tin oxide ceramics. *J. Eur. Ceram. Soc.*, **20**, 297-302 (2000)
